# Kai-Xin-San protects against mitochondrial dysfunction in Alzheimer’s disease through SIRT3/NLRP3 pathway

**DOI:** 10.1186/s13020-023-00722-y

**Published:** 2023-03-14

**Authors:** ShiJie Su, Gongcan Chen, Minghuang Gao, Guangcheng Zhong, Zerong Zhang, Dongyun Wei, Xue Luo, Qi Wang

**Affiliations:** Science and Technology Innovation Center, Guangzhou University of Chinese Medicine, Guangzhou, China

**Keywords:** Kai-Xin-San (KXS), Reactive oxygen species (ROS), Mitochondrial dysfunction, Sirtuins3, NLRP3 inflammasome, Alzheimer’s disease (AD)

## Abstract

**Background:**

Kai-Xin-San (KXS) has been reported to have a good curative impact on dementia. The purpose of the study was to determine whether KXS might ameliorate cognitive deficits in APP/PS1 mice and to evaluate its neuroprotective mechanism.

**Methods:**

APP/PS1 mice were employed as an AD animal model; Aβ_1–42_ and KXS-containing serum were used in HT22 cells. Four different behavioral tests were used to determine the cognitive ability of mice. Nissl staining was utilized to detect hippocampal neuron changes. ROS, SOD, and MDA were used to detect oxidative stress levels. Transmission electron microscopy and Western blot were used to evaluate mitochondrial morphology, mitochondrial division, and fusion state. Western blotting and immunofluorescence identified PSD95, BDNF, NGF, SYN, SIRT3, and NLRP3 inflammasome levels.

**Results:**

The results indicated that KXS protected APP/PS1 mice against cognitive impairments. KXS suppressed neuronal apoptosis and oxidative stress among APP/PS1 mice. KXS and KXS-containing serum improved mitochondrial dysfunction and synaptic and neurotrophic factors regarding APP/PS1 mice. In addition, KXS and KXS-containing serum enhanced mitochondrial SIRT3 expression and reduced NLRP3 inflammasome expression in APP/PS1 mice.

**Conclusion:**

KXS improves cognitive dysfunction among APP/PS1 mice via regulating SIRT3-mediated neuronal cell apoptosis. These results suggested that KXS was proposed as a neuroprotective agent for AD progression.

**Supplementary Information:**

The online version contains supplementary material available at 10.1186/s13020-023-00722-y.

## Introduction

Alzheimer’s disease (AD) is a progressive neurological disorder that causes impairments and memory loss [1]. This debilitating disease exacts a heavy toll on clinically infected persons and family cares, as well as an enormous financial burden on society. The current understanding of AD pathogenic mechanisms is incomplete [2, 3]. The malfunction of clinical trials attacking the amyloid-peptide (Aβ) [4], a pathological hallmark of AD, has highlighted the critical need to investigate alternative features of AD pathology.

Reactive oxygen species (ROS) contribute to AD. Extensive oxidative damage resulting from ROS production is detected within the AD brain [5]. Overproduction of ROS due to depletion of antioxidant enzymes may exacerbate neuronal apoptosis by damaging cellular components such proteins, fats, and DNA. It has been hypothesized that mitochondrial malfunction is an underlying cause of AD and other forms of neurodegeneration that are related to advancing age [6]. Mitochondrial dysfunction had been detected among AD patient’s brains and in mouse models of the disease. This dysfunction characterized by a decline in bioenergetics and ATP synthesis, morphological disturbances, a mitochondrial dynamics imbalance, besides mitochondria redistribution [6]. Mitochondrial dysfunction and ROS generation have been identified as a trigger of neuroinflammation [7–9] through the cGAS-STING pathway and/or the NOD-like receptor-related protein 3 (NLRP3) inflammasome [10]. Mitochondrial damages or generation of mitochondrial ROS (mtROS) are important modulator of NLRP3 activation [11]. Moreover, the NLRP3 inflammasome could be activated by mitochondrial DNA (mtDNA) [12]. Recent findings supposed that the NLRP3 inflammasome is triggered among the of AD brains and MCI patients, and APP/PS1 mice [13]. Reducing Aβ deposition and memory loss protection can be achieved with NLRP3 suppression in APP/PS1 mice [14, 15], which implies NLRP3 inflammasome plays a critical role in AD pathology via regulating neuroinflammation [16]. There are reasons to believe that alleviating oxidative stress, mitochondrial dysfunction and neuroinflammation has the therapeutic effect of improving cognitive impairment and memory dysfunction.

The brain has high levels of sirtuin expression. Evidence suggests that sirtuins play a significant role in protecting neuronal health while aging, and that their levels change with age and are variably expressed throughout various brain regions [17]. Multiple mechanisms linked with AD pathophysiology, as APP processing, Tau protein aggregation, mitochondrial disorders, neuroinflammation, and oxidative damage, could be modulated by SIRTs [18, 19]. Mitochondrial sirtuin 3 (SIRT3), a mitochondrial deacetylase, is essential for energy balance, mitochondrial biogenesis, and oxidative stress regulation [20]. Downregulation of SIRT3 in AD patients’ brains has been documented [21] and experimental AD mouse models [22]. In addition, studies have also shown that Aβ rises Tau by Mediating Sirtuin 3 in AD [23]. Overexpression of SIRT3 decreased Tau acetylation, whereas downregulation of SIRT3 increased Tau acetylation in hippocampal neurons of mice [24]. Furthermore, reduced SIRT3 levels cause p53-mediated mitochondrial malfunction and brain damage in AD [18].

Kai-Xin-San (KXS), a classical Chinese medicinal formula composed of Panax ginseng *C. A. Meyer*, Polygala tenuifolia *Willd.*, Acorus tatarinowii *Schott.* and Poria Cocos (Schw.) *Wolf.* Emerging evidence indicated that KXS has a good curative effect on various neuropsychiatric diseases, especially dementia and depression [25]. Previous research has demonstrated that neuronal apoptosis and tau pathology are both ameliorated in elderly SAMP8 mice by KXS treatment [26]. KXS reduces inflammation, oxidative stress, and neuronal degeneration to mitigate doxorubicin-induced cognitive impairment [27]. KXS contributes to the antidepressant effect by suppressing NLRP3 inflammasome activation and enhancing autophagy [28]. Our previous studies indicated that KXS could ameliorate scopolamine-induced cognitive dysfunction [29]. Additionally, we verified the anti-inflammatory properties of KXS in an AD model [30]. However, the underlying mechanisms of KXS for neuroprotection remain largely unknown. We aimed to explore whether KXS improves AD model learning and memory ability via the SIRT3-dependent pathway. This study employed APP/PS1 double transgenic mice as an AD animal model.

## Material and methods

### Reagents and antibodies

Amyloid beta (Aβ_1–42_) peptide was acquired from (GL Biochem, Shanghai, China). Yuanye Biotechnology procured hexafluoroisopropanol (HFIP). Dimethyl Sulfoxide (DMSO) was imported from (Biomedicals, Santa Ana, USA). 3-(4,5-dimethylthiazol-2-yl)-2,5-diphenyltetrazolium bromide (MTT) was obtained from Sigma Aldrich (Saint Louis, MO, USA). Dulbecco’s Modified Eagle Medium (DMEM), Phosphate Buffered Saline (PBS), Dulbecco’s Modified Eagle Medium/Nutrient Mixture F-12 (DMEM/F-12), Fetal Bovine Serum (FBS), penicillin (100 U/ml) and streptomycin (100 μg/ml) was obtained from Gibco BRL, Gaithersburg (MD, USA). Dye 4,6-diamidino-2-phenylindole (DAPI) was obtained from Roche Diagnosis Co., Ltd. (Shanghai, China). Nissl Staining Solution (C0117) was purchased from the Beyotime Institute of Biotechnology (Jiangsu, China). Total Superoxide Dismutase (T-SOD) Assay Kit (A001-1-1), Malondialdehyde (MDA) Assay Kit (A003-1-1), Reactive oxygen species (ROS) Assay Kit (E004-1-1) were acquired from (Jiancheng Bioengineering, Nanjing, China); NAD+/NADH Assay Kit (S0175, Bbeyotime); Bovine serum albumin (BSA) was purchased from (Roche, Shanghai, China). Primary antibodies: anti-Synaptophysin (ab8049), anti-PSD95 (ab13552), anti-NGF (ab6199), anti-BDNF (ab108319), anti-p-Drp1(ab193216) were obtained from Abcam, Inc; anti-SIRT3 (bs-6105R) were obtained from BIOSS, Inc; anti-NLRP3 (15101S), anti-IL-18 (DF6252), anti-Caspase-1 (AF5418), anti-Cleaved-Caspase 1 (AF4005) and anti-*β*-actin (AF7018) were purchased from Affinity, Inc; anti-IL-1β (12242S), anti-Mitofusin-1 (14,739), anti-Mitofusin-2 (9482), anti-Drp1 (8570) and anti-ASC (67824S) were obtained from Cell Signaling Tech., Inc. Secondary antibodies: Goat Anti-Rabbit IgG H&L (HRP) (ab6721), Goat Anti-Mouse IgG H&L (HRP) (ab6789) and Goat Anti-Rabbit IgG H&L (Alexa Fluor^®^ 555) (ab150078) were obtained from Abcam, Inc.

### Preparation of KXS

Panax ginseng *C. A. Meyer*, Polygala tenuifolia *Willd.*, Acorus tatarinowii *Schott.* and Poria Cocos (Schw.) *Wolf.* (Lot number: 190827, 20191101, C20053007 and 045200303) were purchased from Yuzhou Kaixuan Pharmaceutical Co., Ltd., and Anguo Runde Pharmaceutical Co., Ltd., respectively. Four raw herbs were mixed in a 3:2:2:3 ratio. According to our previous reports, the extraction of KXS and related quality control methods are prepared. The extracts were concentrated, lyophilized, and stored at − 20 °C (Table 1).

**Table 1:** Composition of drugs and their ratio of Kai-Xin-San

Herbal medicine name	Latin name	Ratio
Ren Shen	Panax ginseng *C. A. Meyer*	3
Yuan Zhi	Polygala tenuifolia *Willd*	2
Shi Chang Pu	Acorus tatarinowii *Schott*	2
Fu Ling	Poria Cocos(Schw.) *Wolf*	3

### Animals and drug administration

The Center for Experimental Animals at the Guangzhou University of Chinese Medicine maintained a specified pathogen-free (SPF) environment for male APP/PS1 mice that were 2 months old and age-matched C57BL/6 mice were acquired from the Guangdong Medical Animal Center. The source and specific information of APP/PS1 mice is B6; C3-Tg (APPswe, PSEN1dE9)85Dbo/Mmjax; MMRRC Strain #034829-JAX [31]. All animals were given unrestricted access to food and water, and they were maintained on a light/dark cycle that lasted for 12 h continuously. All experiments were performed with the authorization of the animal ethics committee and according to the Health Guide National Institutes for the Laboratory Animals Care and Use (Bethesda, MD, USA).

APP/PS1 animals were randomly assigned into four groups till all mice reached 9 months of age: APP/PS1 group, APP/PS1 + KXS-L (2.5 g/kg/day) group, APP/PS1 + KXS-M (5 g/kg/day) group and APP/PS1 + KXS-H (10 g/kg/day) group (n = 8). Two groups of C57BL/6 mice were allocated randomly: Control and Control + KXS-H (10 g/kg/day) groups (n = 8–10). KXS were given orally once/day for a month, while mice among Control and APP/PS1 group were given oral gavage with saline.

### Behavioral tests

#### Morris water maze test

The circular pool, which was 120 cm in diameter, was divided into quarters, with a platform measuring 10 cm in diameter set at the bottom of the fourth region level. The mice were tested with the Morris water maze after being treated for 4 weeks. During the adaptive training phase, a 1-min swimming period was provided for the mice. The mice were trained from four different water inlets every day for the following 5 consecutive days. If they could not find the platform within the 60 s, each mice were instructed to stand on the platform for 20 s. The mice will have 60 s of platform-free swimming time to investigate the pool on day 7. All the experimental results will be documented by the SuperMaze^®^ software.

#### Open field test

The open-field test contains a square box (50 × 50 cm) split into 25 squares (16 peripheral and 9 central). The camera system can automatically identify the movement track of the animal and record various activity parameters, including the movement distance of the central area and the time spent in the central area. At the beginning of the experiment, the mice were placed from a corner to the floor. The camera synchronously recorded the movement track and related parameters of the mice within 5 min. Keep the test room quietly. After each mouse was gently placed back in its cage, the chamber was sprayed with 70% alcohol to remove foreign bodies and odors.

#### Novel object recognition test

Before the experiment, each mouse was given three minutes to acclimate to the laboratory setting by moving freely in the empty chamber. In the formal experiment, two identical objects were put into the set position in the chamber. Then the mice were respectively put into the chamber from a fixed corner in the test box for 5 min exploration time. The following day, replace one of the pair of duplicates with a new object, leaving the same position. Mice were put into the laboratory box, and each mouse was given an exploration time of 5 min respectively. The total time exploring new and old objects was recorded as New (N) and Familiar (F), respectively. Novel object preference index = N/(N + F) was taken as the index to judge the ability of mice’s episodic memory. Before each mouse learns, it must first wipe the inside and bottom of the chamber with 70% alcohol and remove animal waste and odors.

#### Elevated zero maze test

Two open arm-areas and two closed arm-areas comprise the elevated zero maze. The diameter of the outer circle is 50 cm, and the diameter of the inner circle is 45 cm. The maze was 50 cm above the ground. During the experiment, the operator placed the mice in the open-arm area with the head facing the inner ring and recorded the animals’ behavior changes for 5 min with the camera system, including the entry time and entry times of the two arms areas. All four paws entered an arm through the central region before recording the experimental parameters. To prevent the experiment from being influenced by the lingering odour of a prior animal, we removed its faeces and doused the track with 70% ethanol before wiping it dry.

### Animal anesthesia and euthanasia

To minimize pain and humanely sacrifice the mice, sodium pentobarbital (50 mg/kg, IP) was used to anesthetize each subject. Random cervical dislocation was used for half of the mice scarification among each group. The hippocampus was quickly removed on ice, cleaned in PBS buffer, and stored at – 80 °C for further detection. PBS was used to perfuse the other anesthetic mice, and then PBS containing 4% paraformaldehyde has been used (PFA). The brain samples were taken from mice and fixed overnight with 4% PFA in PBS for further analysis.

### Nissl stain

4 μm paraffin-embedded brain samples were sectioned, dewaxed, and hydrated. After that, Nissl solution was used to stain brain slides for 10 min. Finally, the slices were sealed by neutral resin after dehydrating, and dimethyl benzene transparency, and a light microscope equipped with LEICA QWin+ was then utilized to evaluate the slides (Wetzlar, Germany).

### Transmission electron microscopy (TEM)

The hippocampal tissue of the mice that had just been sacrificed was cut into small pieces (1 mm × 1 mm × 1 mm) and fixed at 4 °C in 2.5% glutaraldehyde (PBS buffer). Then hippocampal samples were sent to Guangzhou KingMed Center for Clinical Laboratory Co., Ltd. (Guangzhou, China) for transmission electron microscopy. Mitochondrial ultrastructure and shape were assessed by TEM.

### Measurement of oxidative stress

The level of ROS, MDA along with T-SOD activity, and the ratio of NAD+/NADH were determined as per the related protocols of instructions.

### Immunofluorescence stain

The frozen sections of 20 μm were used for immunofluorescence stain. Tissue sections were placed in 0.5%Triton X-100 for permeability at 37 °C for 1 h; 3% catalase was added to the slices to infiltrate the tissues and incubate them for 10 min to eliminate the endogenous oxidase activity of the tissues; 10% goat serum was added to seal at 37 °C for 1 h; primary antibody (SIRT3/NLRP3) was added to the tissues. The slices were stored overnight in a moist box at 4 °C. The fluorescent secondary antibody was incorporated the following day and incubated for 1 h at 37 °C; DAPI dyed the nucleus; A fluorescence microscope (Nikon 80i) was utilized for observation.

### KXS-containing serum preparation

The Animal Center at Guangzhou University provided 20 male SD rats weighing 220 20 g. The experiment was splitted into the normal group (n = 10) and the KXS-containing serum group (n = 10). The KXS-containing serum group was given Kai-Xin-San (10 g/kg) twice for 7 consecutive days. Another 10 rats received an identical dose of saline solution. The related serum was centrifuged at 3000×*g* for 15 min at 4 °C. The upper serum was taken and bathed at 56 °C for 30 min, mixed in the same group. The serum was filtered and inactivated by a 0.22 μm filter and stored at − 20 °C.

### Aβ_1–42_ preparation and cell culture

1.1 mL HFIP was added to 5 mg Aβ_1–42_, mixed and stood at 37 °C for 1 h. each 100 μL dissolved Aβ_1–42_ was added to 22 μL DMSO and 978 μL DMEM/F12 phenol-free red medium. The solution was kept in an incubator at 37 °C for a week. After 7 days, the samples were centrifuged at 14,000×*g* for 30 min at 4 °C, and the 100 mM Aβ_1–42_ supernatant was taken. Briefly, HT22 cells were grown in DMEM supplemented with 10% (v/v) FBS, 100 U/ml of penicillin, and 100 g/ml of streptomycin (Gibco BRL, Gaithersburg, MD, USA). At 37 °C and a constant atmosphere with 5% CO_2_, the cells were kept.

### Cell treatment and cell viability assay

The quantity of crystallization of MTT is inversely proportional to the number of cells within a given concentration range, making the MTT assay a useful indirect indicator of cell viability. HT22 cells were spread in 96-well plate with 5000 cells/well. Following 24 h, Aβ_1–42_ (0 mM, 1 mM, 5 mM, 10 mM, 20 mM, 40 mM, 50 mM) and Blank/KXS-containing serum (10%, 15%, 20%, 25%, 30%) were added. After different intervention times (12 h, 24 h, 36 h, 48 h, 60 h), 10 μL MTT was added to each well for 4 h, 50 μL DMSO were applied to each well. The micro-oscillator vibrated for 10 min so that the light absorption value of each hole was fully determined by the micrometer at the wavelength of 490 nm. The effect of Aβ_1–42_ and the blank/drug-containing serum itself on the activity of HT22 cells was identified to determine the optimal modeling concentration.

### Western blotting analysis

RIPA buffer lysis was used to collect samples of hippocampus or HT22 cells. After centrifuging the lysate for 15 min at 12,000×*g* (4 °C), we retrieved the supernatant. To determine the precise amount of proteins, a measurement was taken using a NanoPhotometer^®^ NP80 (Implen, Germany). SDS-PAGE was used for protein separation, and then the proteins were transferred to a PVDF membrane (Millipore, Billerica, MA). Following a 1-h incubation at room temperature with 5% BSA as a blocking agent, an overnight incubation at 4 °C with primary antibodies specific for target proteins, and a final 1-h incubation at RT with the secondary antibody, membranes were probed with antisera. We utilized the ECL + kit (Applygen, Beijing, China) for detection, and the Bio-Rad Image Lab 5.2.1 software for quantification (Ca, USA).

### Transfection of SIRT3 siRNA

SIRT3 siRNA and negative control siRNA (NC siRNA) sequence was purchased from (Suzhou GenePharma Co., Ltd). Fresh media was plated in six-well plates containing HT22 cells. SIRT3 siRNA or NC siRNA transfection was conducted using the Lipofectamine 3000 kit (Invitrogen, Carlsbad, CA, USA). After a day of incubation for serum-free culture, cells were supplemented with 10 mM Aβ_1–42_ and 20%Blank/KXS-containing serum for 24 h.

### Statistical analysis

We performed our statistical analyses in SPSS 19.0 and GraphPad Prism 5. We utilized one-way analysis of variance with post hoc to analyze the data, and we defined statistical significance at *P* < 0.05. For the statistical description of measurement data, we used mean ± SEM for data that followed a normal distribution, and we used M (P25, P75) for data that did not.

## Results

### KXS protects against cognitive deficit in APP/PS1 mice

**Fig. 1:**
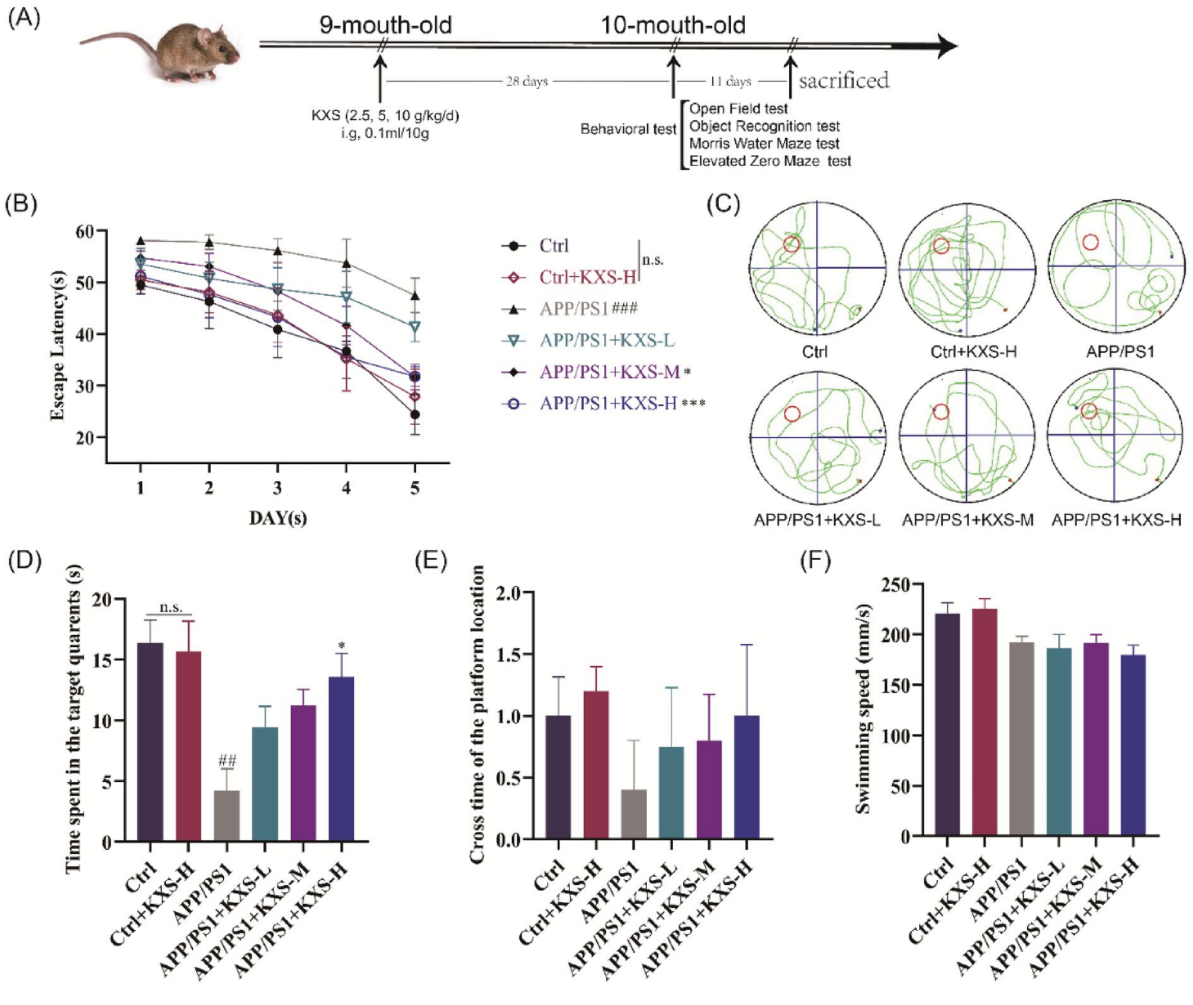
Kai-Xin-San (KXS) protects against cognitive deficit in APP/PS1 mice. Morris water maze test. **A** Experiment schedule; **B** Escape latency training for 5 consecutive days A in PP/PS1 mice; **C** Representative Diagram of swimming paths in the spatial probe trial; **D** Tim spent in the target quadrants; **E** Cross time of the platform location; **F** Swimming speed. KXS-L: 2.5 g/kg/day; KXS-M: 5 g/kg/day; KXS-H: 10 g/kg/day. The experiment data are expressed as means ± SEM. n = 8 in each group. ^#^*P* < 0.05, ^##^*P* < 0.01 vs. Ctrl group; ^∗^*P* < 0.05 vs. APP/PS1 group

**Fig. 2:**
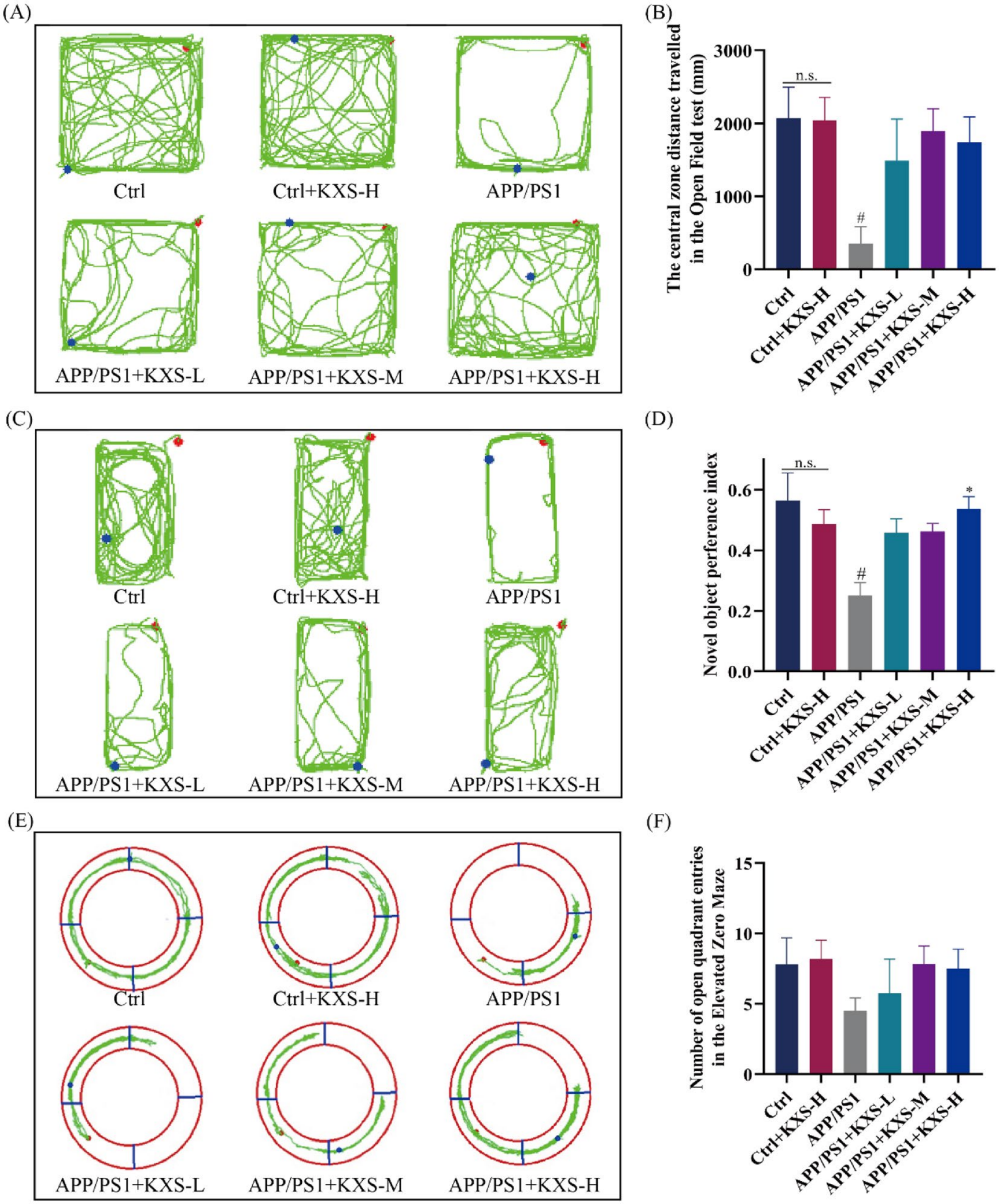
KXS protects against cognitive deficits and anxiety states in APP/PS1 mice. **A** The moving tracks of the Open Filed test; **B** The central zone distance traveled in the Open Filed test; **C** The moving tracks of the New Object Recognition; **D** Novel object preference index; **E** The moving tracks of the Elevated O Maze test; **F** The number of open arm entries in the Elevated O Maze. KXS-L: 2.5 g/kg/day; KXS-M: 5 g/kg/day; KXS-H: 10 g/kg/day. The experiment data are expressed as means ± SEM. n = 8 in each group. ^#^*P* < 0.05 vs. Ctrl group; *P < 0.05 vs. APP/PS1 group

APP/PS1 mice were tested utilizing Morris water maze, open field, new object recognition, and raised O maze to determine the influence of KXS on their learning and memory abilities. Figure 1A showed the experimental schedule on animals. As shown in Fig. 1B, KXS-H (10 g/kg/days) exhibited no discernible impact on C57BL/6 mice’s learning and memory abilities throughout the course of 5 days of escape latency. The ability of 10-month-old APP/PS1 mice to learn and memory was significantly reduced in comparison to control group. The learning and memory abilities of APP/PS1 mice treated with varying dosages of KXS was significantly enhanced and the period required to locate the platform decreased as training days increased (*P* < 0.05, *P* < 0.001). In spatial probe test, APP/PS1 mice treated with KXS-H spent significantly more time on the target platform and crossed it more frequently than untreated mice (*P* < 0.05) (Fig. 1C–E). The swimming speed of APP/PS1 negative mice was slightly faster than APP/PS1 mice, but the difference was not statistically significant (*P* > 0.05) (Fig. 1F). KXS-H (10 g/kg/day) exhibited no significant influence on the behavioral performance of APP/PS1-negative mice in the open field during the new object recognition and raised O maze tests. Compared with C57BL/6 mice, the central zone distance traveled of 10-month-old APP/PS1 mice (Fig. 2A, B), the new object recognition index (Fig. 2C, D) significantly reduced (*P* < 0.05, *P* < 0.05), although open arm entries number is a downward trend, there is no statistical difference (*P* > 0.05) (Fig. 2E, F). However, APP/PS1 mice with different doses of KXS showed various degrees of improvement in related behavioral indicators, especially in the treating with KXS-H of the new object recognition index (*P* < 0.05).

### KXS suppresses neuronal apoptosis and oxidative stress in APP/PS1 mice

**Fig. 3:**
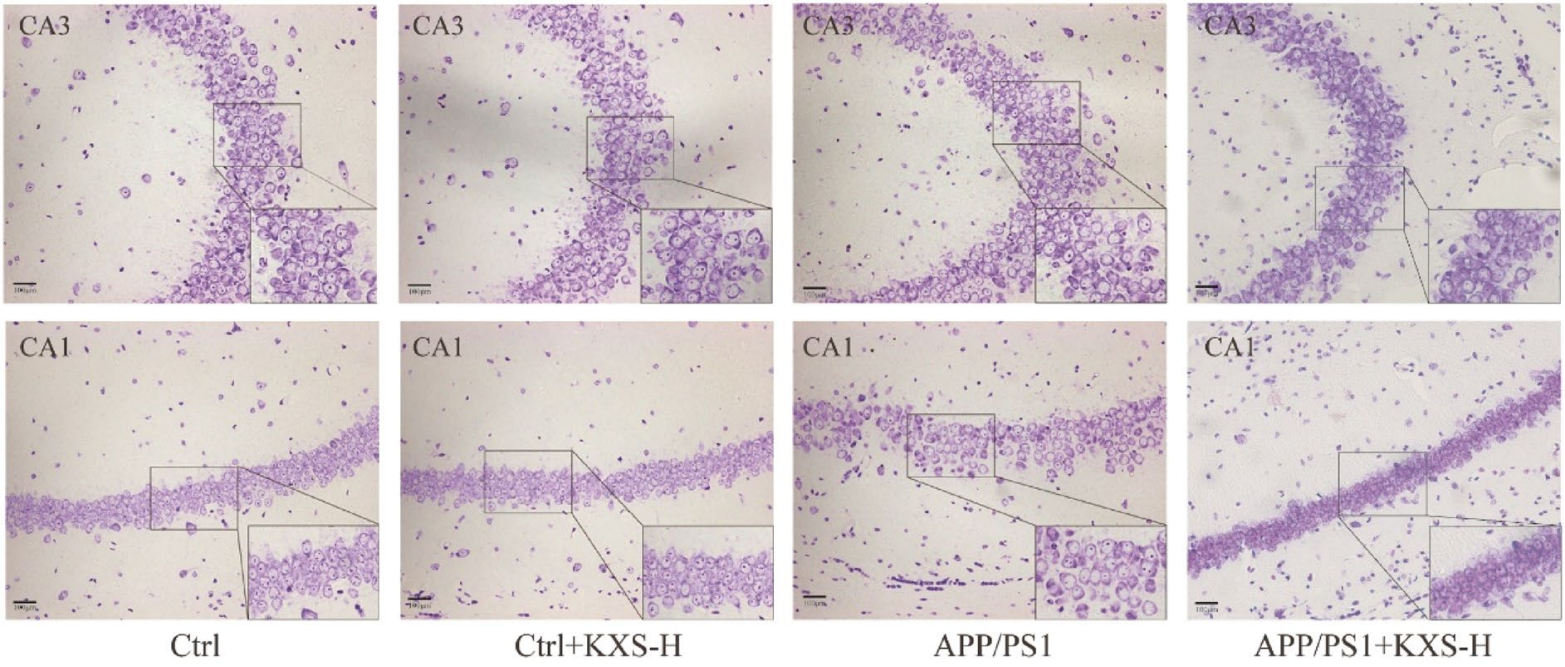
Representative Nissl staining of hippocampal CA1 and CA3 regions of APP/PS1 mice. n = 3 in each group

**Fig. 4:**
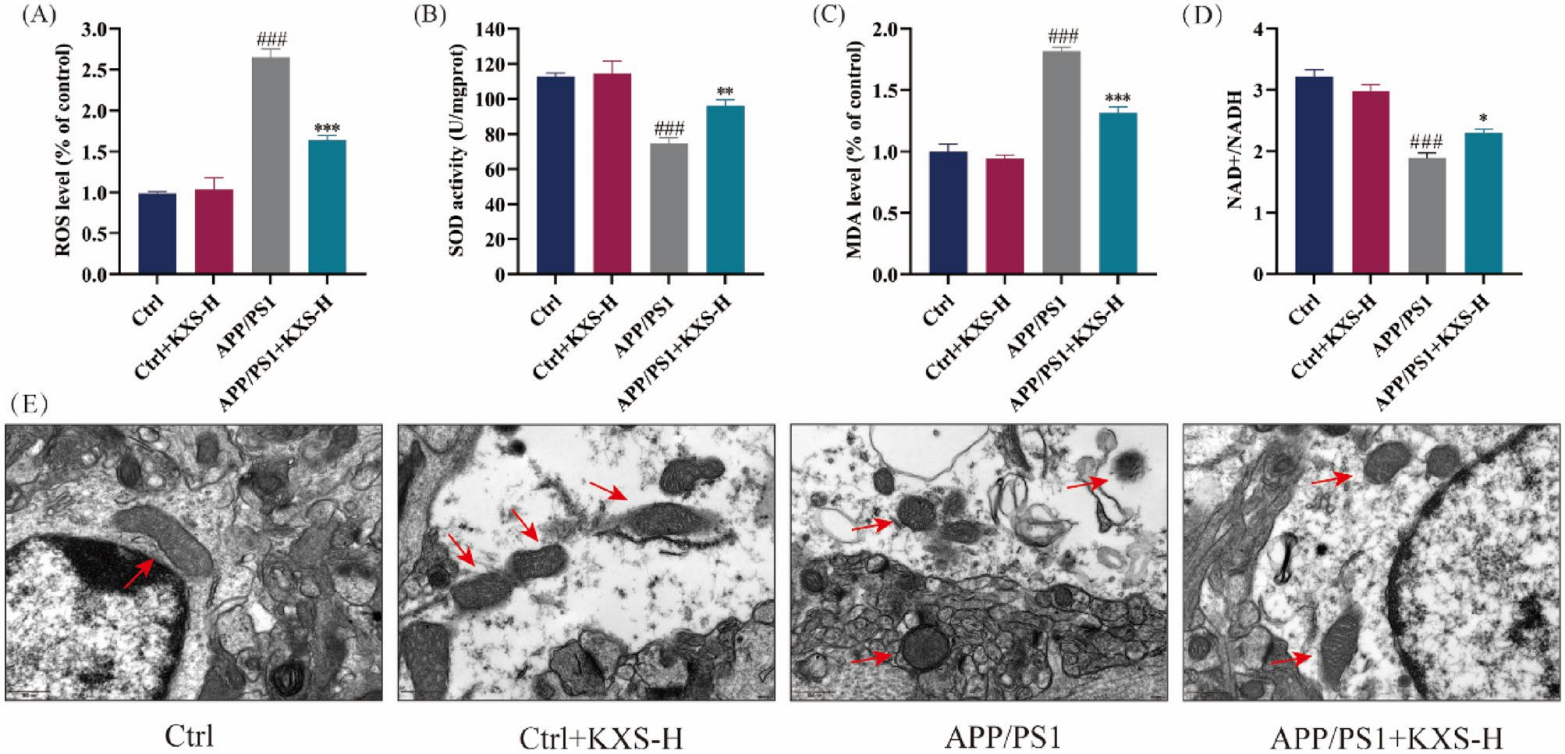
KXS decreases oxidative stress and mitochondrial morphology in hippocampus of APP/PS1 mice. **A** ROS level; **B** SOD activity; **C** MDA level; **D** NAD+/NADH; **E** Representative transmission electron microscope (TEM) photos. KXS-H: 10 g/kg/day. The experiment data are expressed as means ± SEM. n = 3 in each group. ^#^*P* < 0.05, ^##^*P* < 0.01, ^###^*P* < 0.001 vs. Ctrl group; *P < 0.05, **P < 0.01, ***P < 0.001 vs. APP/PS1 group

Nissl staining revealed that KXS-H dosage (10 g/kg/day) administered to C57BL/6 mice in hippocampus CA1 and CA3 regions did not have a significant impact on the number of healthy neurons and neurons that had survived. The number of healthy and surviving neurons in hippocampal CA1 and CA3 regions of 10-month-old APP/PS1 mice significantly reduced, that resulted in typical neuropathy, involving loss of Nissl body and nuclear disappearance in comparison to C57BL/6 mice. However, KXS-H (10 g/kg/day) improved hippocampal neurons’ survival and prevented the loss of neurons in CA1 and CA3 areas and the Nissl bodies in APP/PS1 mice (Fig. 3, Additional file 1: Figure S1). The findings revealed that KXS could prevent hippocampal neurons apoptosis in APP/PS1 mice. Furthermore, to evaluate the improvement influence of KXS on oxidative stress in APP/PS1 mice, ROS, T-SOD, MDA and the ratio of NAD+/NADH were used to detect the hippocampal tissues of APP/PS1 mice. As displayed in Fig. 4A–D, ROS and MDA levels in APP/PS1 mice were significantly higher than those in C57BL/6 mice, whereas T-SOD activity and the ratio of NAD+/NADH were significantly diminished. The findings suggested that KXS-H could decrease ROS and MDA level and raise T-SOD activity and the ratio of NAD+/NADH in hippocampus of APP/PS1 mice.

### KXS improves mitochondrial dysfunction in the hippocampus of APP/PS1 mice

**Fig. 5:**
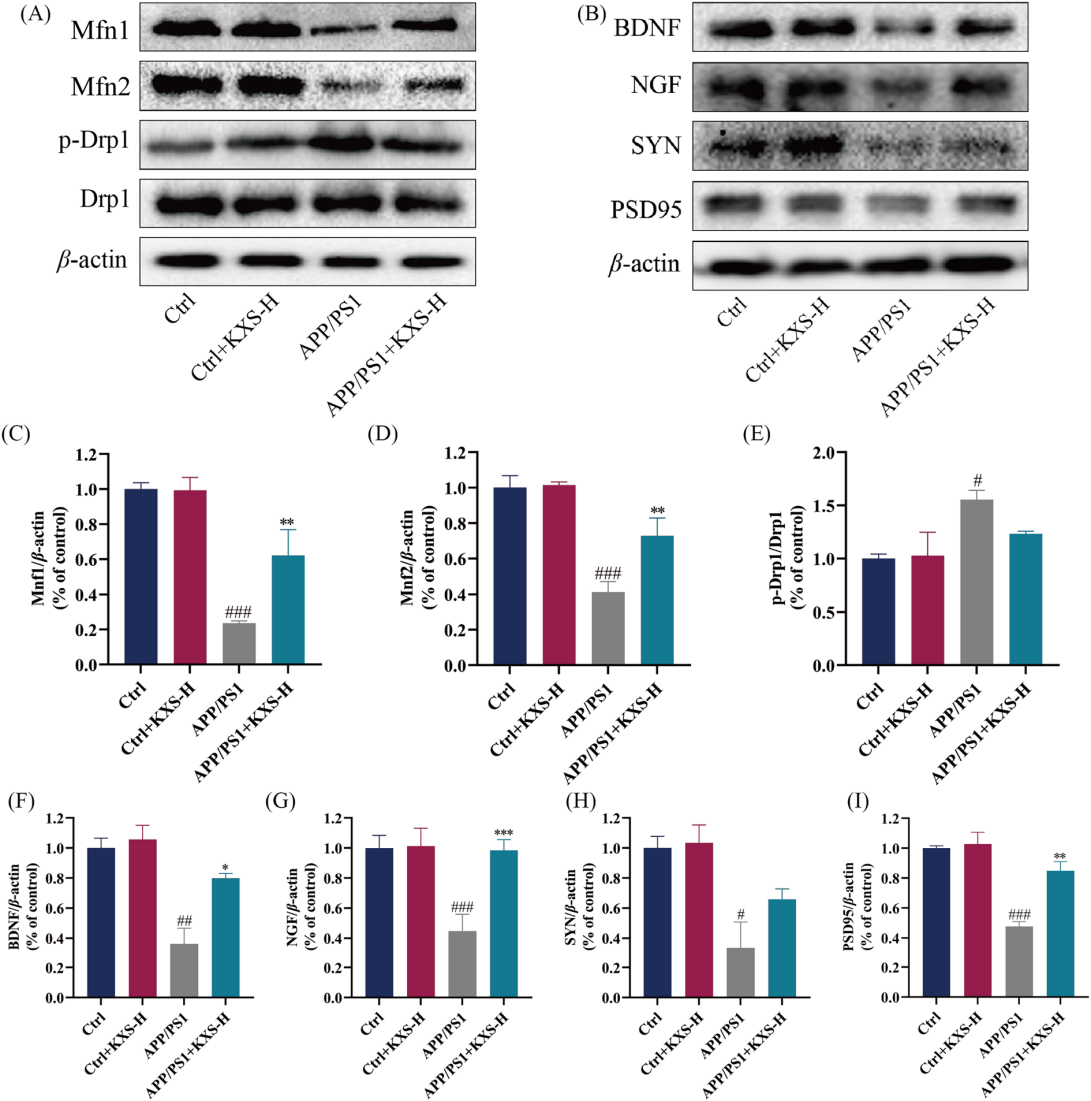
KXS decreases the expression of mitochondrial fission and fusion-related proteins and increases the expression of neurotrophic factors and synaptic proteins in the hippocampus of APP/PS1 mice. **A** Expression of Mfn1, Mfn2, p-Drp1 and Drp1; **B** Expression of BDNF, NGF, SYN and PSD95; **C**–**E** Histogram of relative expression of Mfn1, Mfn2, p-Drp1 and Drp1; **F**–**I** Histogram of relative expression of BDNF, NGF, SYN and PSD95. KXS-H: 10 g/kg/day. The experiment data are expressed as means ± SEM. n = 3 in each group. ^#^*P* < 0.05, ^##^*P* < 0.01, ^###^*P* < 0.001 vs. Ctrl group; *P < 0.05, **P < 0.01, ***P < 0.001 vs. APP/PS1 group

To investigate KXS impacts on mitochondrial morphology of hippocampal neurons in APP/PS1 mice, we conducted TEM to observe mitochondrial morphology (Fig. 4E). The results of TEM exposed that the mitochondrial size of hippocampal neurons in C57BL/6 mice was basically normal, the stromal was uniform, the boundary and structure were clear, the mitochondrial cristae were dense and full, in the shape of a short stick or dumbbell, and no obvious mitochondrial swelling or vacuolar changes were detected. The hippocampus neuron mitochondria of C57BL/6 mice after KXS-H (10 g/kg/day) did not differ significantly from those of control group. In APP/PS1 mice group, the mitochondria of hippocampal neurons were swollen; most of them had swelling and vacuole-like degeneration, the matrix was uneven, the structure was fuzzy, and the swollen mitochondrial cristae had obvious rupture or even swelling rupture. The mitochondria morphology improved after the treatment of KXS-H, and the number of swollen or vacuolated mitochondria was significantly reduced. Additionally, we discovered by Western blot that mitochondrial division and fusion balance was disrupted in 10-month-old APP/PS1 mice KXS-H upregulated the expression of mitochondrial fusion proteins Mfn1 and Mfn2 and downregulated the mitochondrial mitogens Drp1 and p-Drp1, restoring the integrity of the mitochondrial structure (Fig. 5A, C–E). Therefore, we postulated that KXS-H alleviate mitochondrial dysfunction and thereby avoid cognitive impairment in APP/PS1 mice.

### KXS increases the expression of synaptic and neurotrophic factors in the hippocampus of APP/PS1 mice

We identified lower levels of neurotrophic factor (BDNF, NGF, SYN) and synaptic protein (PSD95) in APP/PS1 mice aged 10 months compared to C57BL/6 mice. Nevertheless, the treatment of APP/PS1 mice with KXS-H elevated neurotrophic factor expressions (BDNF, NGF, SYN) and synaptic protein (PSD95) (Fig. 5B, F–I). The results showed that KXS-H had an obvious neuroprotective effect.

### KXS increases the expression of mitochondrial SIRT3 and reduces neuroinflammation in the hippocampus of APP/PS1 mice

**Fig. 6:**
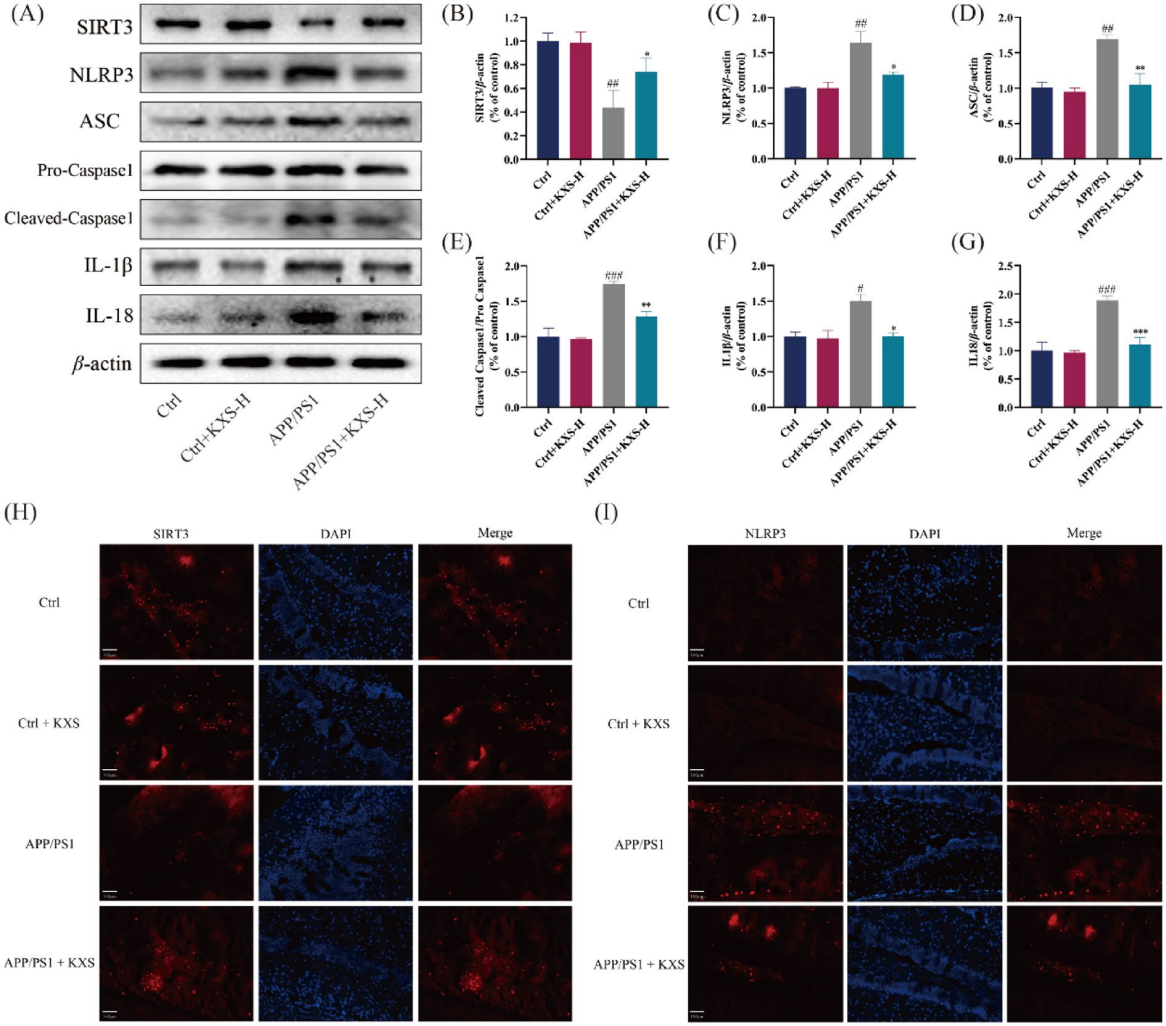
KXS up-regulated the expression of SIRT3 proteins and down-regulated the expression of neurotrophic NLRP3 inflammasome-related proteins in the hippocampus of APP/PS1 mice. **A** Expression of SIRT3 and NLRP3 inflammasome-related proteins; **B** Histogram of relative expression of SIRT3 proteins; **C**–**G** Histogram of relative expression of NLRP3 inflammasome-related proteins; **H** Representative immunofluorescent imaging of SIRT3 (red) and DPAI (blue); **I** Representative immunofluorescent imaging of NLRP3 (red) and DPAI (blue). KXS-H: 10 g/kg/day. The experiment data are expressed as means ± SEM. n = 3 in each group. ^#^*P* < 0.05, ^##^*P* < 0.01, ^###^*P* < 0.001 vs. Ctrl group; *P < 0.05, **P < 0.01, ***P < 0.001 vs. APP/PS1 group

Western blotting (Fig. 6A–G) and immunofluorescence (Fig. 6H–I, Additional file 1: Figure S2) results showed that 10-month-old APP/PS1 mice exposed down-regulated SIRT3 protein expression and up-regulated NLRP3 inflammasome-associated proteins (NLRP3, ASC, pro-Caspase1, cleaved-Caspase1, IL-1β, IL-18), compared with C57BL/6 mice. After KXS-H administration, SIRT3 expression in hippocampus of APP/PS1 mice was up-regulated, while NLRP3 inflammasome-associated proteins expression was down-regulated. These results indicated that KXS-H might improve learning and memory impairment by modifying SIRT3/NLRP3 expression in the APP/PS1 mice hippocampal.

### KXS-containing serum attenuates Aβ_1–42_-induced cytotoxicity in HT22 cells

**Fig. 7:**
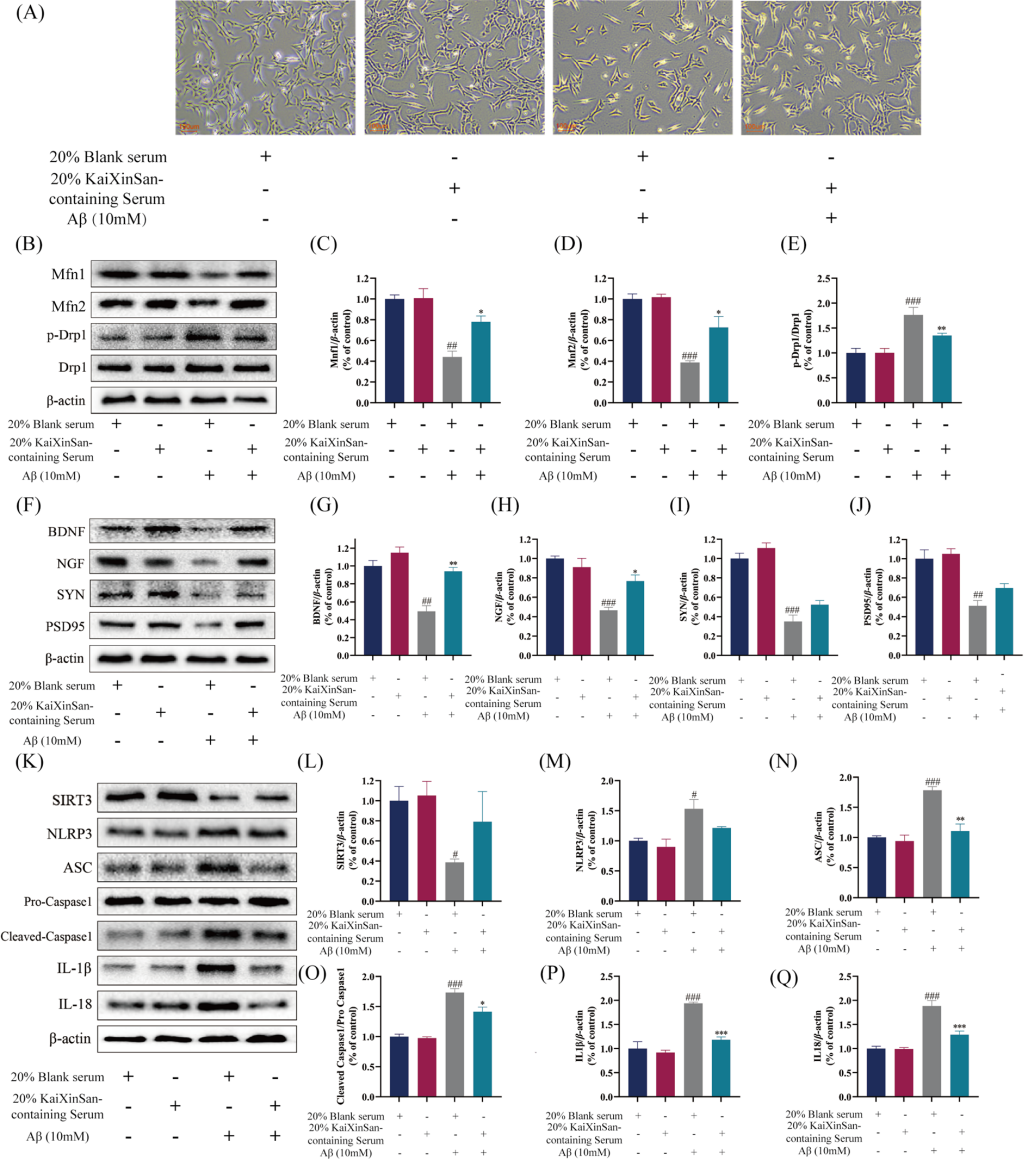
The effect of KXS-containing serum in morphological changes, mitochondrial fission and fusion-related proteins, neurotrophic factors and synaptic-related proteins and SIRT3/NLRP3 pathway in HT22 cells. **A** Morphological changes in HT22 cells treated with Aβ and KXS-containing serum. **B** Expression of Mfn1, Mfn2, p-Drp1 and Drp1; **C**–**E** Histogram of relative expression of Mfn1, Mfn2, p-Drp1/Drp1; **F** Expression of BDNF, NGF, SYN and PSD95; **G**–**J** Histogram of relative expression of BDNF, NGF, SYN and PSD95. **K** Expression of SIRT3 and NLRP3 inflammasome-related proteins; **B** Histogram of relative expression of SIRT3 proteins; **L**–**Q** Histogram of relative expression of NLRP3 inflammasome-related proteins. KXS-H: 10 g/kg/day. The experiment data are expressed as means ± SEM. n = 3 in each group. ^#^*P* < 0.05, ^##^*P* < 0.01, ^###^*P* < 0.001 vs. Ctrl group; *P < 0.05, **P < 0.01, ***P < 0.001 vs. APP/PS1 group

We evaluated the cytotoxicity of KXS-containing serum on Aβ-induced HT22 cells by morphological observation and MTT assay. Preliminary experiments showed that the activity of Aβ_1–42_-induced HT22 cells reduced in a concentration-dependent manner within 1–50 mM and 12–60 h of HT22 cells, and the 10 mM of Aβ-treated HT22 cells showed moderate damage to HT22 cells within 24 h (Additional file 1: Figure S3). Hence, this concentration was utilized in all subsequent experiments. Additionally, 12–60 h of treatment with different doses of KXS-containing serum lowered the activity of HT22 cells in a concentration-dependent manner, and all HT22 cells were moderately damaged by 20% KXS-containing serum (Additional file 1: Figure S4). Therefore, this concentration was used in all further experiments. As shown in Fig. 7A, cell morphology observation showed that 20% KXS-containing serum prevented the death of HT22 cells and reversed the morphological changes of HT22 cells, including cell shape treated with 10 mM Aβ_1–42_. MTT assay results showed that after 24 h of treatment with 10 mM Aβ_1–42_, the activity of HT22 cells decreased, and 20% KXS-containing serum m increased the survival rate of HT22 cells (Additional file 1: Figure S5).

### KXS-containing serum mitochondrial dysfunction and the expression of synaptic and neurotrophic factors

We found that 20% blank serum and 20% KXS-containing serum had no significant influence on mitochondrial division and fusion balance of HT22 cells by western blotting. The mitochondrial division and fusion balance of HT22 cells by 10 mM Aβ_1–42_-induced was destroyed, and the activity of the HT22 cells was impaired. The 20% KXS-containing serum can upregulate Aβ_1–42_-induced mitochondrial fusion proteins Mfn1 and Mfn2 expression and down-regulate the mitochondrial mitogenic proteins Drp1 and p-Drp1 (Fig. 7B–E). In addition, 20% blank serum and 20% KXS-containing serum did not significantly affect neurotrophic factor and synaptic protein expression in HT22 cells. The expressions of 10 mM Aβ_1–42_-induced HT22 cells neurotrophic factor (BDNF, NGF, SYN) and synaptic protein (PSD95) were significantly decreased. However, the expressions of Aβ_1–42_-induced HT22 cell neurotrophic factor (BDNF, NGF, SYN) and synaptic protein (PSD95) significantly increased after administering 20% KXS-containing serum (Fig. 7F–J). The results showed that 20% KXS-containing serum had an obvious neuroprotective effect on Aβ_1–42_-induced HT22 cells.

### SIRT3 is involved in the anti-neuroinflammation effect of KXS-containing serum on HT22 cells

**Fig. 8:**
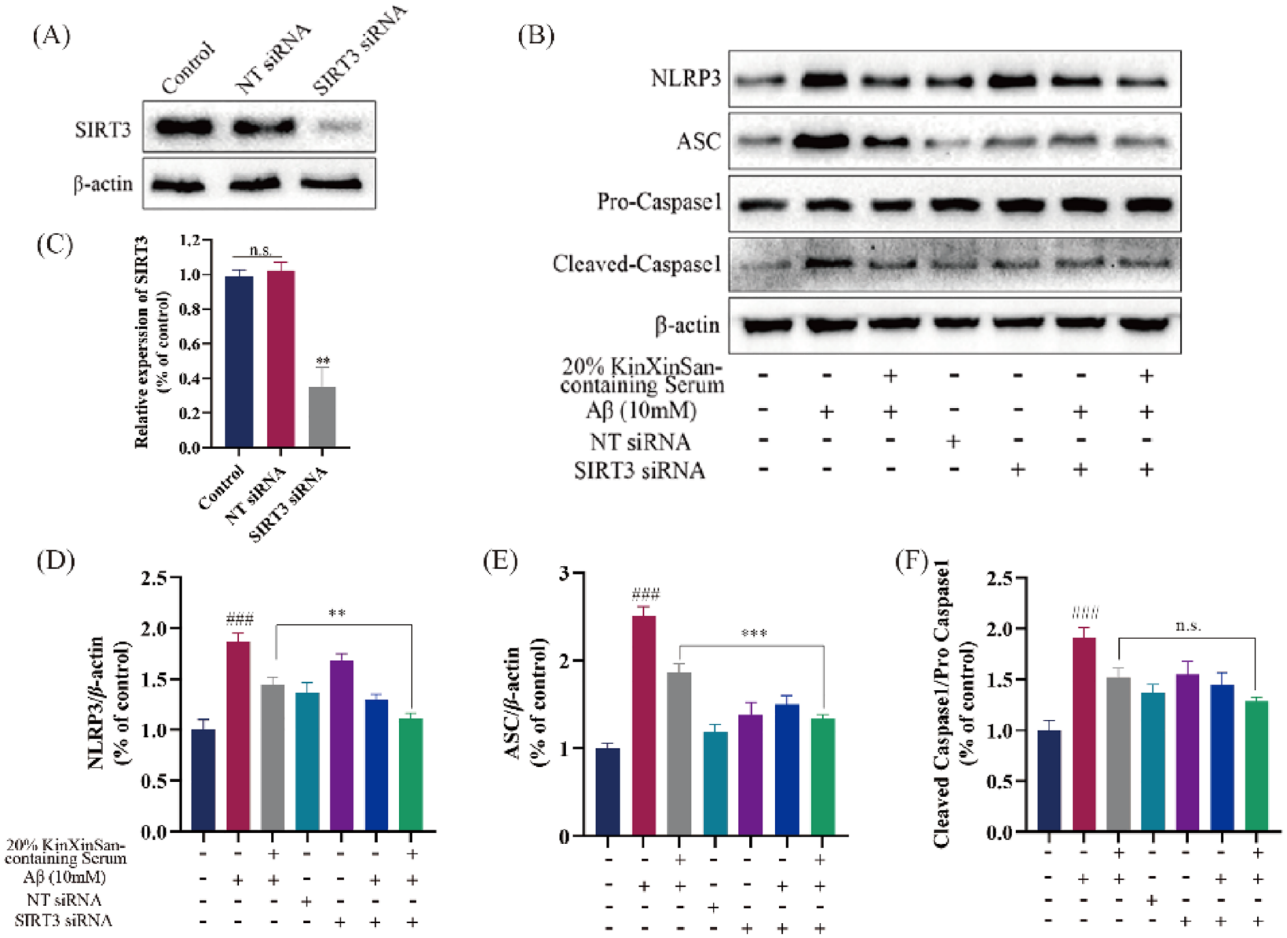
SIRT3 is involved in the antioxidant effect of KXS-containing serum on HT22 cells. **A** The expression of SIRT3; **B** The expression of NLRP3, ASC, Pro-Caspase1 and Cleaved-Caspase1; **C** Histogram of relative expression of SIRT3 proteins; **D**–**F** Histogram of relative expression of NLRP3 inflammasome-related proteins. The experiment data are expressed as means ± SEM. n = 3 in each group. ^###^*P* < 0.001 vs. Ctrl group; **P < 0.01, ***P < 0.001

We studied 20% KXS-containing serum impact on 10 mM Aβ_1–42_-induced HT22 cells in the SIRT3/NLRP3 pathway. Compared with the 20% blank serum or 20% KXS-containing serum, the expression of SIRT3 protein in 10 mM Aβ_1–42_-induced HT22 cells was down-regulated, and NLRP3 inflammasome-associated proteins (NLRP3, ASC, pro-Caspase1, cleaved-Caspase1, IL-1β, IL-18) expression was up-regulated. SIRT3 protein expression was up-regulated and NLRP3 inflammasome-associated protein expression was down-regulated in 10 mM A1–42-induced HT22 cells following treatment with 20% KXS-containing serum (Fig. 7K–Q). To examine SIRT3 function in Aβ-induced HT22 cells treated with 20% serum containing KXS. The indicated plasmids were transfected into HT22 cells, which make the SIRT3 expression was reduced in HT22 cells (Fig. 8A, C). Western blot showed that 20% KXS-containing serum reduced NLRP3 inflammasome activation in HT22 cells. In addition, SIRT3 siRNA transfection further reversed the upregulation of NLRP3 inflammasome treated with 20% KXS-containing serum in HT22 cells (Fig. 8B, D, E). These results indicate that 20% KXS-containing serum plays an anti-neuroinflammatory function by activating SIRT3.

## Discussion

The effects of the Chinese herb Kai-Xin-San on the brain’s memory and learning processes in APP/PS1 double transgenic mice were studied. KXS could improve learning and memory dysfunction in 10-month-old APP/PS1 mice, according to the study’s behavioral tests. Simultaneously, KXS possessed he functions of anti-neuronal apoptosis, anti-oxidant stress, and improvement of mitochondrial dynamics and morphological malfunction properties. Furthermore, KXS could increase the expression of SIRT3 protein and decrease NLRP3 inflammasome-related protein expression in hippocampus. These results suggested that KXS has promise as a treatment for AD.

Cognitive deficits are associated with progressive neuronal apoptosis and synaptic loss in AD [32, 33]. Hippocampal neurogenesis is crucial for cognition, and BDNF and NGF are involved in the neurogenesis process [34]. The consistent expression of synaptic proteins like PSD95 and SYN is necessary for appropriate synaptic function [35]. Enhancing synapse-associated proteins expression has the ability to sustain synaptic connections, which could alleviate memory and cognitive impairment [36]. The results of the Nissl stain and Western blot suggested that KXS could improve hippocampal neurons apoptosis in APP/PS1 mice, by increasing synaptic connections, and upregulating neurotrophic factor levels. The term “oxidative stress” refers to the imbalance between free radical and antioxidant production. Since neurons in the brain generate energy and consume oxygen at rapid rates, they are particularly vulnerable to excessive ROS production and oxidative stress. As neurons are so easily damaged by free radicals, abnormal ROS production and signaling can have far-reaching effects on brain function and behavior [37]. Several studies have provided evidence that oxidative stress is early pathogenesis of AD, which leads to neuronal apoptosis and synaptic loss [38]. Furthermore, oxidative stress can be caused by the free radicals produced by Aβ and tau clumps in AD patients and animal models [39, 40]. Numerous ROS scavengers that could ameliorate oxidative stress-mediated synaptic and cognitive performance in AD patients and animal models have been discovered, given the importance of ROS to the etiology of Alzheimer’s disease [41–43]. Thus, antioxidant treatment is considered a potentially useful strategy for AD prevention and therapeutic management. Our study demonstrated that KXS might enhance SOD activity and reduce ROS and MDA levels. The results suggested that KXS could reduce the ROS level, protecting the function of neuronal cells from the damage caused by excessive ROS.

The activation of neuronal apoptotic cascade, cognitive problems, and behavior functions may be exacerbated by persistent oxidative stress and mitochondrial malfunction. Mitochondria fission and fusion proteins are principally responsible for controlling the dynamics of mitochondria [44]. A variety of human illnesses, as AD and other neurodegenerative diseases, are related with abnormal mitochondrial dynamics and the resulting abnormalities in mitochondrial organization [45]. The fission and fusion mechanisms that directly alter the structure and morphology of mitochondria are the driving behind mitochondrial dynamics. As a result, the structure and function of mitochondria are inextricably intertwined [46]. Large GTPases mediate mitochondrial fission and fusion, with Drp1 being responsible for controlling mitochondrial fission and mitofusin (MFN) being responsible for regulating mitochondrial fusion [47]. There is a noticeable disruption in the balance between mitochondrial fusion and fission in AD neurons. Excessive mitochondrial fission, such as Mfn1, Mfn2, and OPA1, is responsible for impaired mitochondrial function and neuronal death in AD [48]. Downregulation of Drp1 expression decreases mitochondrial fragmentation and improves mitochondrial fusion and mitochondrial function in AD neurons [49]. Our study found that KXS could improve the swelling and rupture of mitochondria in the hippocampus of APP/PS1 mice by blocking excessive mitochondrial fission Drp1 or recruiting fusion MFN1/2. The results indicated that KXS could improve mitochondrial morphological abnormalities and mitochondrial dysfunction in AD models.

It has been demonstrated that oxidative stress and mitochondrial dysfunction are the triggers that activate NLRP3 inflammasomes [10, 50]. Thioredoxin is liberated from TXNIP when oxidative stress rises, making it available to connect with the NLRP3 inflammasome [51, 52]. It has also been proposed that signals originating from mitochondrial malfunction, such as reactive oxygen species (mtROS), oxidized mitochondrial DNA (mtDNA), or the externalization of the phospholipid cardiolipin, induce NLRP3 activation [53]. NLRP3 inflammasomes are essential molecules in neuroinflammation, and Aβ was responsible for AD pathogenesis in AD models [54]. Neuroinflammation is postulated to be a critical component of AD pathogenesis. NLRP3 inflammasome is activated in AD and mild cognitive impairment (MCI) brains and APP/PS1 mice [13]. NLRP3 inflammasome activation leads to Caspase-1-mediated cleavage of the pro-inflammatory cytokines IL-1 and IL-18, which enhance neuroinflammation. NLRP3 deficiency or NLRP3 inhibitor MCC950 substantially attenuates AD phenotypes, involving spatial memory loss, in aged APP/PS1 mice [55, 56]. Interestingly, our experiments demonstrated that KXS could alleviate neuroinflammation mediated by NLRP3 inflammasome activation which improved cognitive impairment in APP/PS1 mice.

Our results shown that KXS can upregulate SIRT3 in APP/PS1 mice hippocampus. SIRT3 is a mitochondrial nicotinamide adenine dinucleotide (NAD+)-dependent deacetylase. SIRT3 is an essential part of different physiological and pathological processes, including energy metabolism [57, 58], neuronal death [59], oxidative stress [60], mitochondrial dysfunction [61], inflammatory [62] and so on. In AD mice models and post-mortem AD patient brains, changes in SIRT3 levels have been detected [22].

Knockout of SIRT3 significantly exacerbated hippocampal neuron death by ROS [63]. SIRT3 overexpression could ameliorate mitochondrial ROS levels and cell apoptosis, which improves ROS detoxification through activation of MnSOD and catalase. Nicotinamide riboside, a SIRT3 agonist, protects transgenic mouse models of AD against cognitive impairment, synaptic degeneration, and neuronal death [64, 65]. Additionally, a lower SIRT3 protein was associated with mitochondrial dysfunction in AD brain [66]. It has been reported that SIRT3 activities were ameliorated by melatonin and icariin administration, resulting in improved mitochondrial function [67]. In addition, SIRT3 inhibits neuroinflammation by removing damaged pro-inflammatory mitochondria and inhibiting the NLRP3 inflammasome [16]. Activating SIRT3 regulation of mitochondrial dysfunction and neuroinflammation leads to ameliorating cognitive decline in mice [21]. In general, our results indicated that KXS has the pharmacological effect of up-regulating the expression of SIRT3 protein. Up-regulating SIRT3 may play a role of anti-oxidative stress, anti-mitochondrial dysfunction, and anti-neuroinflammation, relieving the hippocampal neurons apoptosis and improving cognitive impairment.

To more explore the involvement of SIRT3 in KXS neuroprotective impacts, KXS-containing serum and SIRT3 siRNA were used for cell intervention in our study. In vitro, we also confirmed that KXS-containing serum protects neurotrophic factors and improves mitochondrial dysfunction in the Aβ_1–42_-induced HT22 cells. Furthermore, transfection with SIRT3 siRNA resulted in the reversal of all NLRP3 inflammasome-related proteins. These results indicate that KXS-containing serum may be a probable SIRT3 activator that can preserve neurons from oxidative stress, mitochondrial dysfunction and neuroinflammation mediated neuronal apoptosis.

## Conclusions

**Fig. 9:**
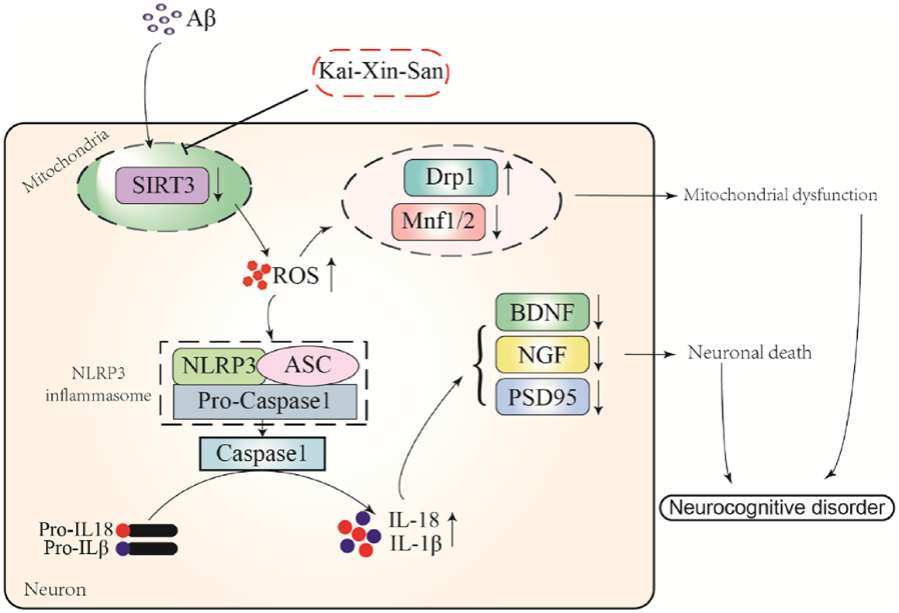
Proposed mechanism of KXS improving cognitive ability in APP/PS1 mice

In conclusion, we found that KXS had neuroprotective characteristics, as it prevented cognitive deficits in APP/PS1 mice. KXS impact on mitochondrial dysfunction and oxidative stress-mediated neuronal cell apoptosis is associated with SIRT3/NLRP3 pathway regulation. KXS has neuroprotective properties that may make it a useful therapeutic Chinese medicine for treating AD (Fig. 9). However, KXS contains several components with complicated network regulatory mechanisms in AD, and the precise molecular targets and processes of KXS in AD have yet to be understood.

## Supplementary Information

**Additional file 1: Figure S1. **The histogram of the number of survival neurons in hippocampal CA1 and CA3 regions of APP/PS1 mice. KXS-H: 10g/kg/day. The experiment data are expressed as means ± SEM. n = 3 in each group. ^###^*P* < 0.001 vs. Ctrl group; ^∗^*P* < 0.05 vs. APP/PS1 group. **Figure S2.** The histogram of immunofluorescent about SIRT3 and NLRP3 in the hippocampus of APP/PS1 mice. n = 3 in each group. ^###^*P* < 0.001 vs. Ctrl group; ^∗^*P* < 0.05 vs. APP/PS1 group. **Figure S3.** The viability of HT22 cells in different concertation Aβ and at different times. n = 3 in each group. *P < 0.05, **P < 0.01, ***P < 0.001 vs. Ctrl group; **Figure S4.** The viability of HT22 cells in different concertation serum (blank serum or KXS-containing serum) and at different times. n = 3 in each group.*P < 0.05, **P < 0.01, ***P < 0.001, ^###^*P <* 0.001. **Figure S5.** The viability of HT22 cells in 20% KXS-concertation serum or 20% blank serum and 10 mM Aβ. n = 3 in each group. ^###^*P* < 0.001 vs. Ctrl group; ∗∗∗*P* < 0.0001 vs. APP/PS1 group.

## Data Availability

The original contributions presented in the study are included in the article/additional material, further inquiries can be directed to the corresponding author.

## Abbreviations

AD: Alzheimer’s disease
Aβ: Amyloid β-protein
SIRT3: Silent information regulator 3
NLR: NOD-like receptor
NLRP3: NOD-like receptor, pyrin domain containing 3
ASC: Apoptosis-associated speck-like protein containing
Caspase: Cysteinyl aspartate specific proteinase
SOD: Superoxide dismutase
MDA: Malondialdehyde
ROS: Reactive oxygen species
Mfn1: Mitofusin1
Mfn2: Mitofusin2
DRP1: Dynamin-related protein 1
IL-18: Interleukin-18
IL-1β: Interleukin-1β
BDNF: Brain-derived neurotrophic factor
NGF: Nerve growth factor
SYN: Synaptophysin
PSD-95: Postsynaptic density protein-95
