# Supplementary material for: Kai-Xin-San protects against mitochondrial dysfunction in Alzheimer’s disease through SIRT3/NLRP3 pathway
Source: Chin Med. 2023 Mar 14;18:26. doi: 10.1186/s13020-023-00722-y (PMC10012453; doi:10.1186/s13020-023-00722-y)
Supplement: Supplementary file 1 — Additional file 1: Figure S1. The histogram of the number of survival neurons in hippocampal CA1 and CA3 regions of APP/PS1 mice. KXS-H: 10g/kg/day. The experiment data are expressed as means ± SEM. n = 3 in each group. ###P < 0.001 vs. Ctrl group; ∗P < 0.05 vs. APP/PS1 group. Figure S2. The histogram of immunofluorescent about SIRT3 and NLRP3 in the hippocampus of APP/PS1 mice. n = 3 in each group. ###P < 0.001 vs. Ctrl group; ∗P < 0.05 vs. APP/PS1 group. Figure S3. The viability of HT22 cells in different concertation Aβ and at different times. n = 3 in each group. *P < 0.05, **P < 0.01, ***P < 0.001 vs. Ctrl group; Figure S4. The viability of HT22 cells in different concertation serum (blank serum or KXS-containing serum) and at different times. n = 3 in each group.*P < 0.05, **P < 0.01, ***P < 0.001, ###P < 0.001. Figure S5. The viability of HT22 cells in 20% KXS-concertation serum or 20% blank serum and 10 mM Aβ. n = 3 in each group. ###P < 0.001 vs. Ctrl group; ∗∗∗P < 0.0001 vs. APP/PS1 group. [file 13020_2023_722_MOESM1_ESM.docx]

**Additional Figure**


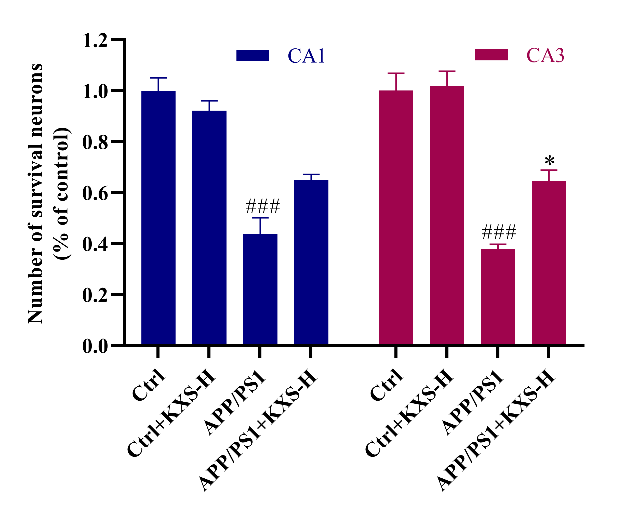

Figure S1 The histogram of the number of survival neurons in hippocampal CA1 and CA3 regions of APP/PS1 mice. KXS-H: 10g/kg/d. The experiment data are expressed as means ± SEM. n=3 in each group.###*P* < 0.001 *vs.* Ctrl group; ∗*P* < 0.05 *vs.* APP/PS1 group.


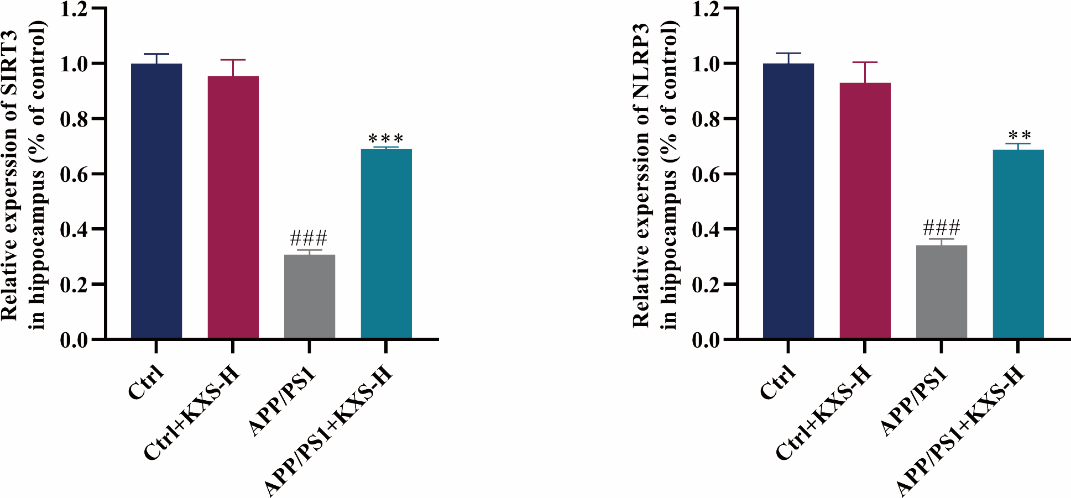


Figure S2 The histogram of immunofluorescent about SIRT3 and NLRP3 in the hippocampus of APP/PS1 mice.n=3 in each group.###*P* < 0.001 *vs.* Ctrl group; ∗*P* < 0.05 *vs.* APP/PS1 group.


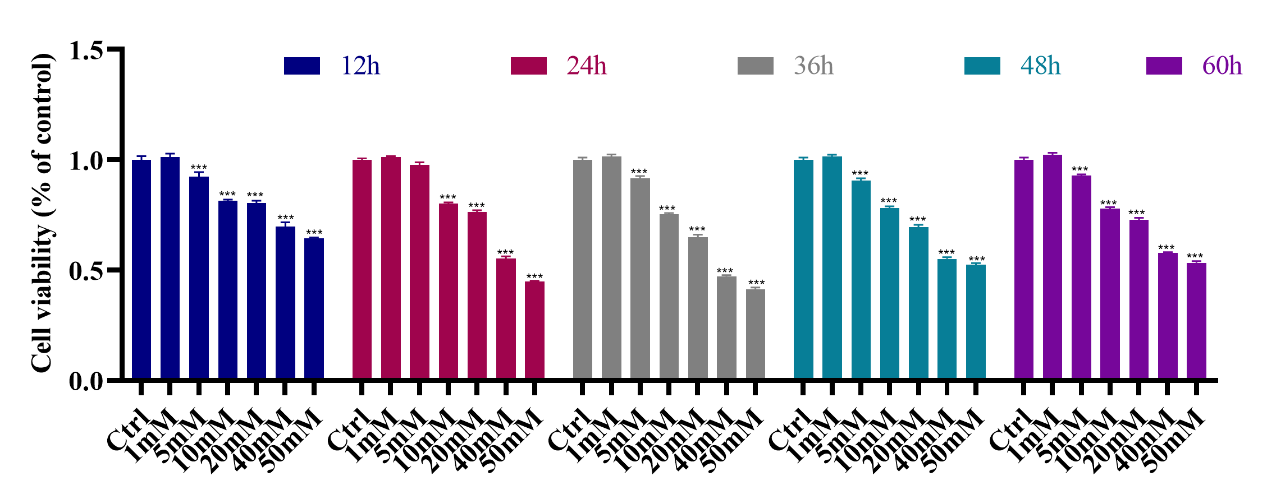


Figure S3 The viability of HT22 cells in different concertation Aβ and at different times.n=3 in each group. **P* < 0.05，***P* < 0.01, ****P* < 0.001 *vs.* Ctrl group;


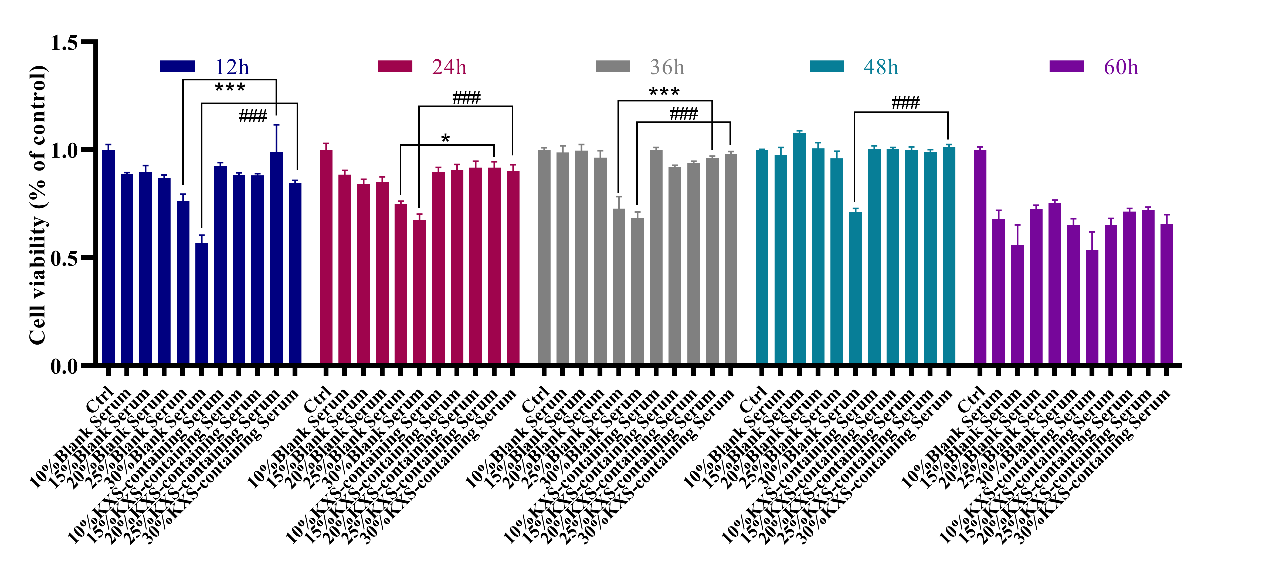


Figure S4 The viability of HT22 cells in different concertation serum (blank serum or KXS-containing serum ) and at different times. n=3 in each group.**P* < 0.05，***P* < 0.01, ****P* < 0.001*, ###P < 0.001*


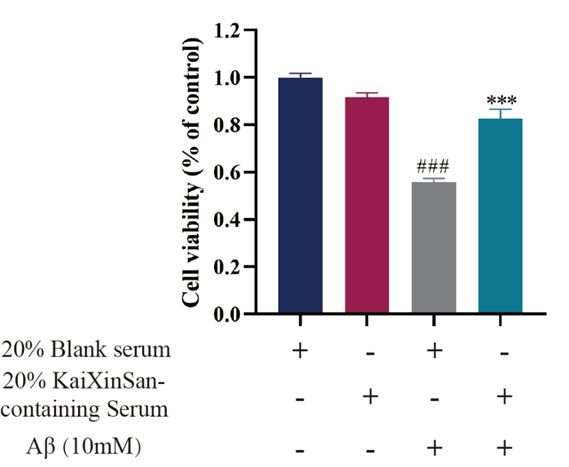


Figure S5 The viability of HT22 cells in 20% KXS-concertation serum or 20% blank serum and 10mM Aβ. n=3 in each group.###*P* < 0.001 *vs.* Ctrl group; ∗∗∗*P* < 0.0001 *vs.* APP/PS1 group.
